# Rapamycin: Drug Repurposing in SARS-CoV-2 Infection

**DOI:** 10.3390/ph14030217

**Published:** 2021-03-05

**Authors:** Jiri Patocka, Kamil Kuca, Patrik Oleksak, Eugenie Nepovimova, Martin Valis, Michal Novotny, Blanka Klimova

**Affiliations:** 1 Institute of Radiology, Toxicology and Civil Protection, Faculty of Health and Social Studies, University of South Bohemia Ceske Budejovice, 37005 Ceske Budejovice, Czech Republic; toxicology@toxicology.cz; 2 Biomedical Research Centre, University Hospital, 50003 Hradec Kralove, Czech Republic; 3 Department of Chemistry, Faculty of Science, University of Hradec Kralove, 50003 Hradec Kralove, Czech Republic; patrik.oleksak@uhk.cz (P.O.); eugenie.nepovimova@uhk.cz (E.N.); 4 Department of Neurology, Charles University, Faculty of Medicine and University Hospital Hradec Kralove, 50003 Hradec Kralove, Czech Republic; martin.valis@fnhk.cz (M.V.); m.novas82@gmail.com (M.N.); blanka.klimova@uhk.cz (B.K.)

**Keywords:** COVID-19, SARS-CoV-19, rapamycin, sirolimus, mTOR inhibitor

## Abstract

Since December 2019, SARS-CoV-2 (COVID-19) has been a worldwide pandemic with enormous consequences for human health and the world economy. Remdesivir is the only drug in the world that has been approved for the treating of COVID-19. This drug, as well as vaccination, still has uncertain effectiveness. Drug repurposing could be a promising strategy how to find an appropriate molecule: rapamycin could be one of them. The authors performed a systematic literature review of available studies on the research describing rapamycin in association with COVID-19 infection. Only peer-reviewed English-written articles from the world’s acknowledged databases Web of Science, PubMed, Springer and Scopus were involved. Five articles were eventually included in the final analysis. The findings indicate that rapamycin seems to be a suitable candidate for drug repurposing. In addition, it may represent a better candidate for COVID-19 therapy than commonly tested antivirals. It is also likely that its efficiency will not be reduced by the high rate of viral RNA mutation.

## 1. Introduction

According to the officially available data, for the first time in December 2019, a new threat to humanity appeared in China in Wuhan. It is a serious acute respiratory disease SARS-CoV-2 (COVID-19), in which the causative agent—a new coronavirus 2—was subsequently identified [1]. In the modern world, COVID-19 soon became a worldwide pandemic with enormous consequences for human health and the world economy. According to the Johns Hopkins University dashboard (as of 23 February 2021), the total number of confirmed cases worldwide was 111,890,818; 2,478,717 deaths and 63,133,922 recovered [2].

There is only one drug in the world that has been approved or authorized in some countries for treating of COVID-19—remdesivir [3]. On the other hand, the recent published guidelines from the World Health Organization (November 2020) do not recommend the use of remdesivir for the treatment of COVID-19 due to uncertain effectiveness [4]. Vaccination is another option in the fight against COVID-19. There are several COVID-19 vaccines for which certain national regulatory authorities have authorized the use (as of 23 February 2021; Table 1). A positive fact is that vaccination is already underway worldwide; however, there are essential limits in its manufacturing and distribution. It should be noted that many scientists assume that, like most other vaccines, COVID-19 vaccines will not be 100% effective [5].

Drug repurposing is a promising strategy how to get an old well-known molecule for a new indication. Many drugs approved for other uses have already been tested in humans. Therefore, detailed information is available on their pharmacology, formulation and potential toxicity. This increasingly used process reduces the time frame, decrease costs and improve success rates, especially in the situation where the epidemic continues rapidly and infected and dead people increase rapidly. Currently, a large number of reviews can be found in the literature that deal with the repurposing of various molecules. Some of them remain only as suggestions in publications; however, many of them are currently undergoing real clinical testing. Repurposed drugs under clinical investigation are generally aim at two targets: directly at SARS-CoV-2 virus (e.g., hydroxychloroquine, chloroquine, remdesivir, favipiravir, lopinavir/ ritonavir, ribavirin); or targeting the host immune system (e.g., thalidomide, human immunoglobulin, heparin, corticosteroids, tocilizumab, sarilumab, canakinumab, anakinra, gimsilumab, baricitinib, ruxolitinib, nivolumab) [7,8,9,10,11]. A recently published study by Tian et al. (2021) emphasizes the use of tocilizumab in prolonging survival in patients with severe COVID-19. This molecule could be a new strategy for COVID-19-induced cytokine release syndrome [12]. 

Effective drug repurposing must also consider the structure of the virus itself. Thus, the following proteins appear to be key: the RNA-dependent RNA polymerase (RdRp) and the nucleocapid protein. RdRp is the core component and plays a central role in COVID-19 replication and transcription cycle. Two nucleoside analogs targeting this protein (the influenza drug favipiravir and the experimental Ebola virus drug remdesivir) are currently being evaluated in clinical trials for the treatment of COVID-19 [13,14,15]. The nucleocapsid (N) protein of coronavirus is a multifunctional RNA-binding protein and forms a helical filament structure that is required for the assembly of viral genomic RNA into a ribonucleoprotein complex. The packaging of the viral genomic RNA regulates viral replication/transcription and modulates infectious cell metabolism. Zidovudine triphosphate (an anti-HIV agent) is a potential inhibitor of the N-terminal domain of COVID-19-protein based on docking and simulation analysis. It should be considered for experimental validations [16,17]. 

A completely different (natural) approach to the possible fight against COVID-19 infection was used by Roviello and Roviello [7]. They suggest that evergreen Mediterranean forests and shrubland plants might serve as protection of southern population and provision of dietary sources of bioactive compounds. The study revealed a potential anti-COVID-19 activity in laurusides, which are unexplored glycosides from bay laurel. 

In this study, we focused on reviewing the current literature that would provide information for the potential repurposing of the mammalian target of rapamycin (mTOR) inhibitor rapamycin based on its known molecular mechanism and targeting [8,9,10]. 

## 2. Materials and Methods

**Figure 1:**
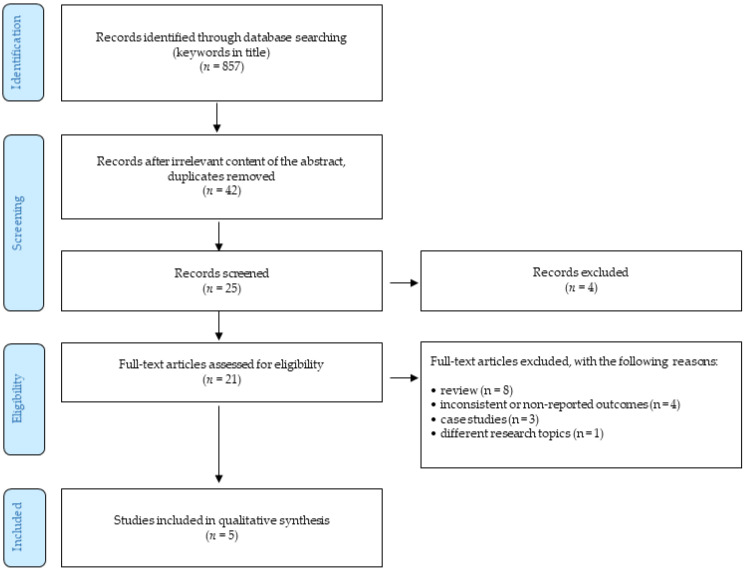
Selection workflow.

The authors performed a systematic literature review of available studies on the research describing rapamycin in association with COVID-19 infection. The methodology follows the Preferred Reporting Items for Systematic Reviews and Meta-Analysis (PRISMA) guidelines (Figure 1). The end of the search period is limited by 31 January 2021. The research studies were selected on the basis of the keywords, such as “rapamycin, sirolimus, mTOR inhibitor, COVID-19, SARS-CoV-19” found in the world’s acknowledged databases Web of Science, PubMed, Springer and Scopus. The terms used were searched using AND to combine the keywords listed and using OR to remove search duplication where possible. In addition, a backward search was also performed, i.e., references of the detected studies were evaluated for relevant research studies that authors might have missed during their search. In addition, a Google search was conducted to identify unpublished (gray) literature. The authors performed an independent quality assessment of these studies. The authors selected these basic quality criteria using the Health Evidence Quality Assessment Tool for review articles. The primary outcome of this review was to explore if there is any possibility to repurpose rapamycin into new indication—COVID-19 treatment or prevention.

Altogether, 857 studies were identified in all these databases. After removing duplicates and titles/abstracts unrelated to the research topic, 42 English-written studies remained. Of these, only 21 articles were relevant for the research topic. These studies were investigated in full and they were considered against the following inclusion and exclusion criteria:

- only peer-reviewed English-written full-text journal articles were involved;
- the time of publishing the article was limited by 31 January 2020.

The exclusion criteria were as follows:

- reviews;
- case studies;
- the articles focusing on different research topics;
- outcomes were not reported or were inconsistent.

Considering the above-described criteria, five articles were eventually included in the final analysis.

## 3. Results and Discussion

**Figure 2:**
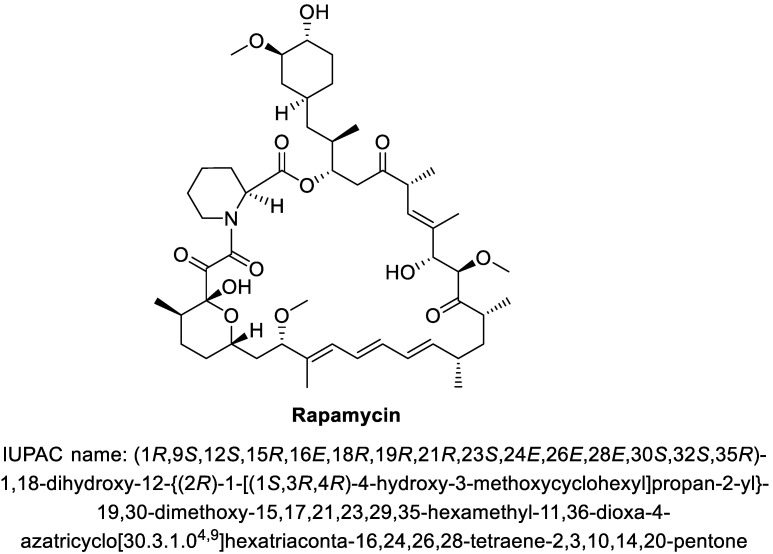
The structure of rapamycin.

Rapamycin (syn. sirolimus, Figure 2) is a macrocyclic lactone, present in soil actinomycete *Streptomyces hygroscopicus* that was found in a sample of mud collected on Easter Island called Rapa Nui [11]. In 1991, the immunosuppressive activity of rapamycin was discovered and it became its most important property used in pharmacology. Rapamycin inhibits the mTOR, protein synthesis and prevents the activation of lymphocytes by blocking mTOR enzyme [12,13]. It also inhibits the expression of proinflammatory cytokines, such as IL-2, IL-6 and IL-10 and suppresses cytokine storm [14]. Rapamycin interrupts the T cell cycle during their transition from G1 to S phase by inhibiting the interleukin-mediated signal [1]. Rapamycin also can inhibit the proliferation of vascular wall smooth muscle cells, which are not affected by other immunosuppressants [15].

Rapamycin was approved by the FDA in September 1999 as an immunosuppressive agent indicated for the prophylaxis of organ rejection in patients receiving renal transplants and have been used safely for decades. Rapamycin has also anticancer activity which was defined in the 1990s and it led to the development and application of semi-synthetic rapamycin analogues (rapalogs) with superior characteristics for oncology (e.g., everolimus, temsirolimus) [16,17]. These facts make rapamycin a suitable candidate for drug repurposing [18,19]. On one hand, rapamycin is not a virustatic agent, but on the other hand, by focusing on host factors (not on the virus itself), it may represent a better candidate for COVID-19 therapy than commonly tested antivirals. It is also likely that its efficiency will not be reduced by the high rate of viral RNA mutation [20].

**Figure 3:**
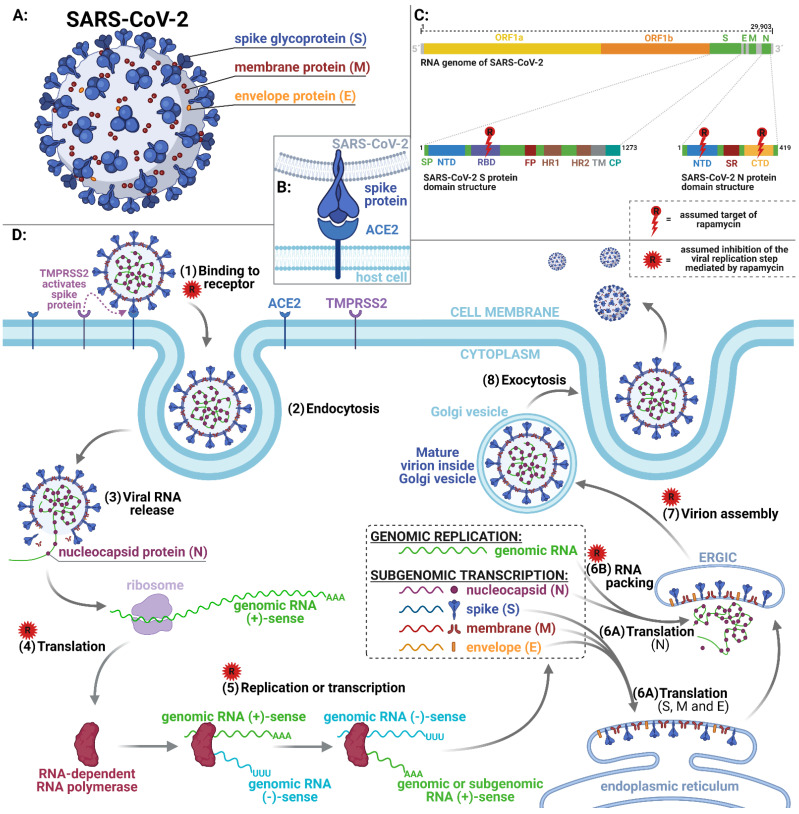
(**A**): Structure of SARS-CoV-2 with structural proteins. (**B**): Detail of SARS-CoV-2 spike protein interaction with ACE2 receptor of the host cell. (**C**): Top: Genome structure of the SARS-CoV-2 involves two genes ORF1a (yellow) and ORF1b (orange) encoding 16 non-structural proteins. Genes encoding four main structural proteins—spike (S), membrane (M), envelope (E) and nucleocapsid (N) are colored green. Bottom left: Major structural domains of SARS-CoV-2 S protein—signal peptide (SP), N-terminal domain (NTD), receptor-binding domain (RBD), fusion peptide (FP), heptad repeat regions 1 (HR1) and 2 (HR2), transmembrane (TM), cytoplasmic tail region (CP). Bottom right: Major structural domains of SARS-CoV-2 N protein—NTD, serine-arginine (SR)-rich domain, C-terminal domain (CTD). Rapamycin´s target domains are highlighted. (**D**): Simplified lifecycle of SARS-CoV-2: (**1**) TMPRS2 receptor activates spike glycoprotein of SARS-CoV-2 toward binding to ACE2 receptor on surface of the host cell. (**2**) Endocytosis of SARS-CoV-2 into the host cell. (**3**) Release of the viral RNA. (**4**) Viral RNA is translated into RNA-dependent RNA polymerase (RdRp). (**5**) RNA-dependent RNA polymerase synthetizes (−)-sense genomic RNA, a template for synthesis of (+)-sense genomic or subgenomic RNA. (**6A**) Translation of the viral structural protein N is performed in cytoplasm, while proteins S, M and E are translated at endoplasmic reticulum. (**6B**) Viral RNA and N protein form RNA-N complex. (**7**) Endoplasmic reticulum-Golgi intermediate complex (ERGIC) with inserted S, M and E proteins and RNA-N complex interact to assembly of the virion. (**8**) Mature virion is released from the host cell via exocytosis. Assumed rapamycin-mediated inhibitions are highlighted. Figure was created with BioRender.com.

Mathematical calculations, computer modeling and other similar approaches represent the most modern methods usable for drugs repurposing. Zhou et al. (2020) presented an integrative, drug repurposing methodology, implementing a systems pharmacology-based network medicine platform, quantifying the interplay between the human coronaviruses–host interactome and drug targets in the human protein–protein interaction network. Thanks to phylogenetic analyses, it was found that the envelope and nucleocapsid proteins of COVID-19/SARS are two evolutionarily conserved regions, having the sequence identities of 96% and 89.6%, respectively, compared to SARS. Using network proximity analyses of drug targets, an interesting drug combination—rapamycin plus dactinomycin—was discovered. The targets of the drugs both hit the human coronaviruses–host subnetwork [21]. Ahamad et al. [18] used multiple computational approaches to explore the potential mechanisms of binding and inhibitor activity of several compounds toward C-terminal domain (CTD) of N protein. Among the drugs, rapamycin displayed the second highest binding affinity (−8.91 kcal/mol) [22]. Moreover, Tatar et al. [23] investigated 34 existing antiviral compounds in docking study on the N protein of SARS-CoV-2. Rapamycin has shown the best binding affinity (−11.87 kcal/mol). However, in contrast to the previous study, Tatar et al. revealed interaction of rapamycin with residues (Lys65, Phe66, Pro67, Arg68, Gly69, Gln70, Ile84, Pro122, Tyr123, Gly124, Ala125, Asn126, Ile130, Ile131, Trp132, Val133, Ala134, Thr135, Glu136, Gly137, Ala138, Asn140) located on N-terminal domain (NTD) of the N protein. This protein of the SARS-CoV-2 virus is necessary for viral RNA replication and transcription. Another important function of N protein is to promote correct viral RNA folding and formation of ribonucleoprotein complex. In addition, the interaction of N protein with M protein drive virion assembly. The mentioned functions of N protein in combination with computed rapamycin’s binding affinity represent a promising strategy for the inhibition of SARS-CoV-2 (Figure 3). Another computational analysis with the goal to find new drugs candidates, based on their ability to modulate oppositely the transcriptional profiles, were published by Fagone et al. [24]. The transcriptomic profile of primary human lung epithelium infected by COVID-19, focusing on the most relevant pathways modulated during the infection, were studied. This analysis shows that the mTOR inhibitor, rapamycin, may be a candidate drug for use in COVID-19 patients, which is in agreement with the data by Zhou et al. [21]. The aims of the study of Gates and Hamed [25] were to identify off-label drugs that may have benefits for the COVID-19 disease pandemic. They present a novel ranking algorithm called CovidX to recommend the existing drugs for potential repurposing and validate the literature-based outcome with drug knowledge available in clinical trials. The CovidX algorithm was based upon a notion that we called “diversity.” A diversity score for a given drug was calculated by measuring how “diverse” the drug is, using various biological entities (regardless of the cardinality of actual instances in each category). The algorithm identified 30 possible drugs and authors chose the top 10 candidates and one of them was rapamycin. Finally, Kalhor et al. [20] used a structure based on virtual screening of the FDA databases where several lead drugs were discovered based on the ACE2-binding pocket of COVID-19 S protein (Figure 3). Then, binding affinity, binding modes, critical interactions and pharmaceutical properties of the lead drugs were evaluated. The conclusion is that rapamycin (as well as other molecules) represented the most desirable features and can be a possible candidate for COVID-19 therapies. A suitable target for inhibition of viral replication is represented by RdRp, an enzyme catalyzing RNA replication from RNA template. Among evolutionary distant RNA viruses, RdRp is a high conserved enzyme. The structure of RdRp contains two channels linked at the position of active site. Specific RdRp inhibitors, such as remdesivir and sofosbuvir, are approved for the treatment of SARS-CoV-2 and hepatitis C virus (HCV) infections, respectively. The inhibition of RdRp results in reduced viral replication. Therefore, RdRp is considered as an attractive antiviral target [26]. Pokhrel et al. [27] performed an in silico virtual screen of selected FDA-approved drugs against the conserved targets in RdRp of SARS-CoV-2. Among the evaluated drugs targeting RNA tunnel of RdRp, rapamycin belongs to the top three compounds. 

In the literature, there are several reviews where we can find information about rapamycin and its possible use during the COVID-19 infection. All of them are just theoretical and are based on pharmacology knowledge of its mechanism or based on analogy with treatments of other similar viral infections in humans or animals. The authors Husain and Byrareddy [7] reviewed the potential use of rapamycin from many points of view. They concluded that the use of rapamycin can help to control viral particle synthesis, cytokine storms and contributes to fight the disease by its anti-aging and anti-obesity effects. Maiese [21] in the review showed that mTOR pathways in conjunction with AMPK may offer valuable targets to control cell injury, oxidative stress, mitochondrial dysfunction and the onset of hyperinflammation, a significant disability associated with COVID-19. Zhavoronkov [22] points out that the COVID-19 infection rates, severity and lethality are substantially higher in the population aged 60 and older. The author discusses this topic from the point of age and some geroprotectors, such as rapamycin and its analogues. mTOR inhibitors (e.g., rapamycin) in preventing the severity of COVID-19 were reviewed in an article by Zheng et al. [23]. The authors highlighted an unmet clinical need to find an effective way to prevent the occurrence of severe illness as severe acute respiratory syndrome COVID-19. Cytokine storms and highly pathogenic human coronaviruses should be taken into consideration [28]. A mechanistic review of inflammatory pathways in COVID-19 was published by Yarmohammadi et al. [24]. One of the crucial roles represents mTOR. Thus, mTOR inhibitors in limiting the proliferation of memory B cells and T-cell responses, patients treated with mTOR inhibitors are expected to a reduced early production of cross-reactive antibody, less antibody-dependent enhancement and fewer severe symptoms in COVID-19 [29]. Ramaiah [25], with reference to other publications, summarized a potential of rapamycin to inhibit COVID-19 infection and replication in human lung cells (e.g., inhibit MERS activity or gives cross-strain protection against influenza infection) [30,31]. Finally, Sargiacomo et al. [27] hypothesized that three FDA-approved drugs (azithromycin, doxycycline and rapamycin) behave as inhibitors of protein synthesis and experimentally have been shown to reduce inflammation and viral replication. Mechanistically, this is because cytokines and viruses are both made of proteins. Both use the cellular ribosomes for protein translation. Inhibiting virus production should help to clinically reduce viral transmission to other patients.

**Figure 4:**
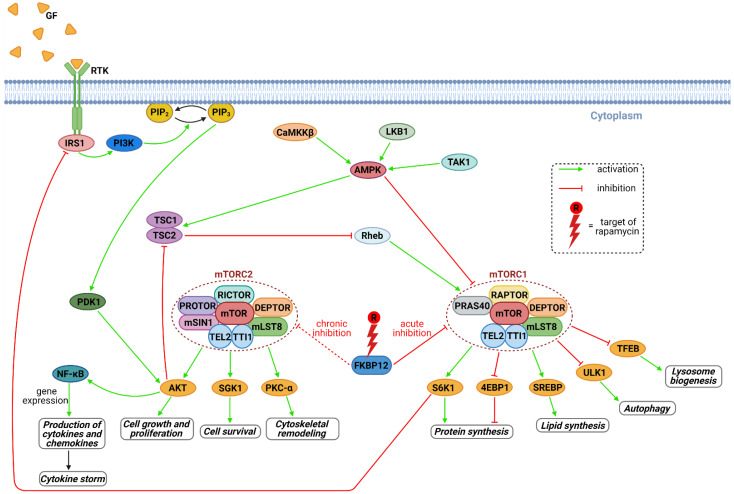
The mTOR signaling pathway and its important regulatory functions are shown. Activated mTORC1 results in increased protein synthesis via downstream effectors S6K1 and 4EBP1. AKT-mediated activation of NF-κB increases gene expression followed by cytokine and chemokine production. Rapamycin acts as the inhibitor of mTORC1 (acute inhibition) and mTORC2 (chronic inhibition) that is crucial for affecting of the downstream pathway. Abbreviations: 4EBP1, eukaryotic translation initiation factor 4E-binding protein 1; AKT, protein kinase B; AMPK, 5’-adenosine monophosphate-activated protein kinase; CaMKKβ, Ca^2+^/calmodulin-dependent protein kinase kinase-β; DEPTOR, DEP domain-containing mTOR-interacting protein; FKBP12, FK506-binding protein of 12 kDa; GF, growth factors; IRS1, insulin receptor substrate 1; LKB1, liver kinase B1; NF-κB, nuclear factor kappa-light-chain-enhancer of activated B cells; mLST8, mammalian lethal with SEC13 protein 8; mSIN1, mammalian SAPK interacting protein 1; PDK1, 3-phosphoinositide-dependent protein kinase 1; PI3K, phosphatidylinositol 3-kinase; PIP_2_, phosphatidylinositol (4,5)-bisphosphate; PIP_3_, phosphatidylinositol (3,4,5)-trisphosphate; PKC-α, protein kinase C alpha; PRAS40, proline-rich AKT substrate of 40 kDa; PROTOR, protein observed with rictor; RAPTOR, regulatory associated protein of mTOR; Rheb, ras homolog enriched in brain; RICTOR, rapamycin-insensitive companion of mTOR; RTK, receptor tyrosine kinase; S6K1, ribosomal protein S6 kinase 1; SGK1, serum- and glucocorticoid-induced kinase 1; SREBP, sterol regulatory element binding protein 1; TAK1, TGF-β activated kinase 1; TEL2, telomere maintenance 2; TFEB, transcription factor EB; TSC1 and 2, tuberous sclerosis complex 1 and 2; TTI1, TEL2-interacting protein 1; ULK1, Unc-51 like autophagy activating kinase. Figure was created with BioRender.com.

Rapamycin is immunosuppressant with well-known mechanisms of action which involve inhibiting of mTOR kinase. mTOR and more specifically a protein complex mTORC1 formed by mTOR, plays a key role in viral replication (Figure 4) [32]. Therefore, rapamycin has been evaluated in the presence of several different viruses and different conditions. Kindrachuk et al. [28] described in vitro experiment, where rapamycin was shown to affect PI3K/AKT/mTOR pathway, which inhibited MERS infection by 61% at 10 μM concentration. This result provides a strong evidence for a critical role for mTOR in MERS-CoV infection. In a presence of severe influenza viruses in murine models, combination of rapamycin and oseltamivir have shown inconsistent effects [33,34]. On the other hand, Wang et al. [31] showed in a group of patients (*n* = 38) with severe H1N1 pneumonia, early adjuvant treatment with corticosteroids and an mTOR inhibitor was associated with improvement in outcomes, such as hypoxia, multiple organ dysfunction, virus clearance and shortened liberation of ventilator and ventilator days.

On one hand, the inhibitory effect of rapamycin on cell proliferation may help to control viral particle synthesis, cytokine storms and contributes to fight the disease by its anti-aging and anti-obesity effects [9]. On the other hand, the inhibition of mTORC1 promotes the autophagy of macrophages (Figure 4); mTOR inhibitors also suppress early B-cell production and decrease the populations of antigen-specific memory B cells. Therefore, it can be expected that patients with COVID-19 infection treated with mTOR inhibitors are anticipated to have a reduced early cross-reactive antibody production and thus less antibody-dependent enhancement. Thus, mTOR inhibitors could act as a double edge sword in patients with COVID-19 [35].

At the time of writing this article (14 February 2021), ClinicalTrials.gov listed 4 interventional studies that included a rapamycin treatment arm in presence of COVID-19 infection. Three of them are at the recruiting stage or earlier (Table 2; NCT04482712, NCT04341675, NCT04461340). One of them was withdrawn with the explanation that the study population was not regularly admitted to hospital and approaches have shifted away from repurposing old drugs (NCT04371640). **NCT04482712** is a single center, double-blind, placebo-controlled, randomized clinical trial in which each participant, after admission to hospital with a diagnosis of mild to moderate COVID-19 infection, will be administered either a dose of 1 mg rapamycin or the placebo daily. Each subject will receive the assigned treatment until hospital discharge or death. Evaluations will be performed at the beginning of the clinical trial and then daily up to 4 weeks. The main objective of **NCT04341675** study is to determine if treatment with rapamycin can improve clinical outcomes in hospitalized patients with COVID-19. The investigators will employ a randomized, double blind, placebo-controlled study design. Subjects will be randomized to receive rapamycin or placebo. Rapamycin will be given as a 6 mg oral loading dose on day 1 followed by 2mg daily for a maximum treatment duration of 14 days or until hospital discharge (whichever happens sooner). Chart reviews will be conducted daily to determine changes in clinical status, concomitant medications and laboratory parameters. Study specific biomarkers will be measured at baseline and then at days 3, 7 and 14. **NCT04461340** study is single-blinded randomized clinical trial in which participants will be randomly assigned to one of the study groups using block randomization. Patients will receive rapamycin (oral dose of 6 mg on day 1 followed by 2 mg daily for 9 days) plus national standard of care therapy against COVID 19. The study planned to illustrate the efficacy and safety of rapamycin as an adjuvant agent to the standard treatment protocol against COVID-19 infection.

It would be useful to mention one interesting case report of a 13-year-old child with multiple comorbidities who acquired COVID-19 5 years post-renal transplantation in the United States. Maintenance immunosuppression consisted of rapamycin (4 mg/day) and mycophenolate (500/250 mg). The symptoms were fever, cough, rhinorrhea and hypoxemia. He did not require intensive care unit care or ventilation. He had an excellent clinical recovery within 4 days and was able to be discharged home.

Finally, it should be noted that rapamycin treatment is not without risk. The most common (>30%) adverse reactions are: peripheral edema, hypertriglyceridemia, hypertension, hypercholesterolemia, creatinine increased, abdominal pain, diarrhea, headache, fever, urinary tract infection, anemia, nausea, arthralgia, pain and thrombocytopenia. Increased susceptibility to infection and the possible development of lymphoma and other malignancies may result from immunosuppression by rapamycin. Physicians should avoid a concomitant use of rapamycin with potent inducers or inhibitors of CYP3A4 that may decrease or increase blood levels. Rapamycin should be used during pregnancy only if the potential benefit justifies the potential risk to the embryo/ fetus. Dosage should be reduced in patients with hepatic impairment [19].

## 4. Conclusions

In the light of the presented findings, rapamycin seems to be a suitable candidate for drug repurposing. In addition, it may represent a better candidate for COVID-19 therapy than commonly tested antivirals. It is also likely that its effectiveness will not be reduced by the high rate of viral RNA mutation. Hopefully, the findings from the ongoing open clinical trials of rapamycin for the treatment of COVID-19 infection will reveal its successful efficacy.

## Figures and Tables

**Table 1:** Summary of the three most common vaccines worldwide (according to who.int) [6].

Vaccine Type	Developer/Name	Phase 3 Trials	Number of Countries with Status Approved
RNA	BioNTech/Pfizer/BNT162b2	NCT04368728 NCT04713553	57
Non replicating viral vector	Oxford/AstraZeneca/AZD1222	CTRI/2020/08/027170 ISRCTN89951424 NCT04536051 NCT04516746 NCT04540393 2020-001228-32 NCT04400838	46
RNA	Moderna/mRNA-1273	NCT04649151 NCT04470427	37

**Table 2:** Summary of open clinical trials of rapamycin for treatment of COVID-19 infection (according to clinicaltrials.gov, accessed on 14 February 2021).

NCT Number	NCT04482712	NCT04341675	NCT04461340
Status	Not yet recruiting	Recruiting	Recruiting
Start	January 2021	24 April 2020	15 August 2020
Title	Effects of mTOR Inhibition with Sirolimus (RAPA) in Patients with COVID-19 to Moderate the Progression of ARDS	Sirolimus Treatment in Hospitalized Patients with COVID-19 Pneumonia	Efficacy and Safety of Sirolimus in COVID-19 Infection
Phase	1/2	2	2
Number of subjects	20	30	40
Age	60+	18+	18+
Planned outcomes	Survival rate; Change in Clinical Status assessed by the WHO scale; Change in Clinical Status assessed by the NIAID scale; All-cause mortality; duration of ECMO; Duration of supplemental oxygen; Length of hospital stay; Length of time to SARS-CoV2 negativity.	Proportion of patients who are alive and free from advanced respiratory support measures at day 28; Proportion of patients who require escalation in care; Change over time in study-specific biomarkers (LDH, Ferritin, D-dimer, lymphocyte count); Proportion of patients surviving to hospital discharge; Drug safety profile; Duration of advanced respiratory support; Duration of hospital stay; Time from treatment initiation to death; Time to resolution of fever; Proportion of patients who require initiation of off-label therapies;	Time to clinical recovery; Viral clearance; Radiological lung extension; drug adverse events; 28 day mortality; Intensive care unit admission rate; Duration of hospital stay; Duration from hospitalization to discharge
Location	San Antonio, TX, USA	Chicago, IL, USA; Cincinnati, OH, USA	Faculty of Medicine, Alexandria university, Egypt
Last update	30 November 2020	20 May 2020	9 September 2020
